# Fitness center use and subsequent achievement of exercise goals. A prospective study on long-term fitness center members

**DOI:** 10.1186/s13102-022-00400-w

**Published:** 2022-01-13

**Authors:** Liv Riseth, Tom Ivar Lund Nilsen, Torunn Hatlen Nøst, Aslak Steinsbekk

**Affiliations:** 1 Department of Public Health and Nursing, Norwegian University of Science and Technology, Post box 8905, 7491 Trondheim, Norway; 2 3T-Fitness Center, Vestre Rosten 80, 7075 Tiller, Norway; 3 Clinic of Anesthesia and Intensive Care, St. Olavs Hospital, Trondheim University Hospital, Trondheim, Norway; 4 Digital Health Care Unit, Norwegian Center for E-Health Research, Tromsø, Norway; 5 Department of Mental Health, Faculty of Medicine and Health Sciences, Norwegian University of Science and Technology, Trondheim, Norway; 6 Norwegian Advisory Unit on Complex Symptom Disorders, St. Olavs Hospital, Trondheim University Hospital, Trondheim, Norway

**Keywords:** Fitness center, Goal achievement, Visits, Fitness trainer, Group activity and fitness center use

## Abstract

**Background:**

Knowledge on the relationship between fitness center use and long-term members’ subsequent goal achievement is limited. Therefore, the aim was to investigate the prospective association between the use of fitness centers during 18 months and subsequent self-reported goal achievement among long-term members.

**Methods:**

This was a registry- and survey-based longitudinal study of 2851 people who had been members at a Norwegian fitness center chain for more than two years. Fitness center use from December 2016 to June 2018 was obtained from registry data. Subsequent goal achievement was measured in a survey in June 2018, assessed by a 1–100 visual analogue scale, and a score between 0 and 50 was defined as low goal achievement.

**Results:**

Visiting the fitness center frequently and regularly, and having frequent group activity bookings were associated with higher subsequent self-reported goal achievement. Participants with fewest visits (1–57 days) during 18 months were more likely to report low goal achievement than participants with most visits (118–543 days) (OR = 8.5; 95% CI 6.3–11.4). Fitness trainer bookings was not clearly associated with subsequent goal achievement.

**Conclusions:**

Frequent and regular long-term fitness center use were associated with higher subsequent self-reported goal achievement.

## Background

Despite overwhelming evidence of physical activity’s beneficial effect on health and well-being [1], the proportion of the adult population who reaches WHO's [2] recommended level of at least 150 min of moderate or 75 min of vigorous physical activity throughout the week is low [3]. The same contradiction is also reflected in the use of fitness centers. Although members of fitness centers intend to use the centers regularly [3, 4], a substantial proportion of members has infrequent and irregular use of the facilities [5]. A large proportion also terminates the membership after a short period [6].

Increasing physical activity and reducing the proportion of people who are physically inactive are significant public health priorities in many countries [8]. To achieve this, knowledge of factors that can motivate people to become and maintain physically active as recommended is therefore warranted [9]. Studies have reported that satisfaction with own goal realization can motivate people to maintain physically active over time [9, 10]. On the other hand, low goal achievement has been associated with negative emotions, reduced self-efficacy, and perceptions of failure that may reduce participation in physical activity [11]. Preventing low motivation and poor goal achievement could therefore be key factors to promote regular and sustained physical activity.

Fitness centers are essential arenas for physical activity in many countries [12]. They offer various exercise options in a safe environment, they facilitate social interaction, and the members often have access to qualified exercise guidance and coaching [13, 14]. Thus, use of such exercise facilities could motivate members to regular and sustained physical activity and aid people to reach their exercise goals.

Therefore, the aim was to investigate the prospective association between the use of fitness centers during 18 months and subsequent self-reported goal achievement among people who had been members at a fitness center for two years or more.

## Methods

This was a registry- and survey-based longitudinal study with membership registry data from December 1st 2016 to May 31st 2018, and survey data collected in June 2018. The "Strengthening the Reporting of Observational Studies in Epidemiology" guidelines were consulted for the reporting of the study [15].

The study was conducted within the setting of a fitness center chain (www.3T.no) in Central Norway. Twelve of 3T’s fitness centers are located in the city of Trondheim, which had approximately 205,000 inhabitants in 2020, and these centers had around 40,000 members at the time of data collection. All centers have a primary workout area, and most of the centers have group classes, fitness trainers, personal trainers, saunas, and member lounges with simple café services. Members can book a free-of-charge session with a fitness trainer to get help and guidance and a personalized exercise program, or choose to pay for a personal trainer for closer follow-up and guidance. Group activities in this chain are also numerous and various, including yoga, stretching, water gymnastics, spinning, strength training and aerobics, lasting from 20 to 90 min.

### Participants and procedures

The inclusion criteria were all adult members, aged 18 years or older at the time of survey distribution, who were registered with an e-mail address, been a paying ordinary member for a minimum of the two previous years and who allowed linkage to their membership data on use of the fitness center. Members with free or employee contracts, who were younger than 18 years of age, or whose memberships had not lasted 2 years were excluded.

3 T’s head office sent an email to all 15,273 eligible members with information about the study and a link to the survey. The landing page for the survey included additional information about the study. Members who were willing to participate could tick two boxes: one confirming that they agreed to participate, the other confirming that they consented to having their membership data on the use of fitness centers be linked to their survey record. A reminder was sent to those who had not answered four days after the first request.

### Data collection

#### Fitness center use

Data on the use of fitness centers was collected from the membership registry, including timestamps for visits, bookings of group activity, and fitness trainer bookings. The number of days with visits during 18 months were categorized into thirds (low (1–57), medium (58–117), and high (118–543)), as well as a separate category for those with zero visits. Similarly, participants were categorized into thirds based on number of group activity bookings (low (1–26), medium (27–80), and high (81–550)), as well as a fourth category with zero bookings. Fitness trainer bookings were categorized into none, one, and two or more bookings. To measure regularity in fitness center visits during the 18-month period, members were classified into three groups based on the number of three-month periods in which they had visited the fitness center at least once: ((1) all six periods, (2) four and five periods, and (3) less than four periods).

#### Self-reported goal achievement

Self-reported goal achievement was measured on a visual analogue scale (VAS) using the question "On a scale from 0 to 100, to what degree do you experience reaching your exercise goals at the fitness center?" (0 [zero] = to a very little extent, 100 = to a very large extent). The distribution of score values was left-skewed, with most people reporting high goal achievement values (median = 71, interquartile range 57–80). For the purpose of the linear regression analyses that compared mean score values between categories of the use of fitness centers, the goal achievement variable was log-transformed after inverting the measurement scale (i.e., higher score value on the inverted VAS indicates lower goal achievement), so that a VAS score of 80 was inverted to 20. To avoid missing data when log-transforming zero, 1 (one) was added to all score values. For logistic regression analyses, we constructed a binary variable where the participants were classified into low and high goal achievement based on the 20th percentile of the distribution of score values. Participants below the 20th percentile (i.e., VAS 0–50) were classified as having low goal achievement, and those on the 20th percentile and above (i.e., VAS 51–100) were classified as having high goal achievement.

#### Demographic data

The survey provided information on sex (male or female), age in years (< 20, 20–29, 30–39, 40–49, 50–59, 60–69 or ≥ 70), highest completed level of education (compulsory, middle, and higher) and employment (full-time work, part-time work, student, or not working due to occupational rehabilitation, unemployment or being laid off, disability benefits, being retired or other).

#### Statistical analyses

Descriptive characteristics of the study population are presented as proportions within the four categories of days with visits. To obtain inferential statistics, we first used linear regression to compare mean goal achievement score between categories of the four variables representing long-term use of the fitness center (i.e., days with visits, three-month periods with visits, group activity bookings, and fitness trainer bookings). For all comparisons, participants with most frequent visits/bookings constituted the reference group. As described above, the scores for goal achievement were first inverted and then log-transformed. The results of the linear regression analyses are therefore presented as the ratio of geometric means with 95% confidence interval (CI) for each category, relative to the refence group. Secondly, we used logistic regression to estimate odds ratio (OR) with 95% CI of poor goal achievement. Similar to the linear regression analyses, participants with most frequent visits/bookings constituted the reference group for all comparisons. All associations were adjusted for age (< 30, 30–39, 40–49, 50–59, 60–69 and ≥ 70 years), higher education (yes or no), and sex (male, female). We used Stata version 17 (StataCorp LLC, College Station, TX, USA) for all analyses.

## Results

**Fig. 1:**
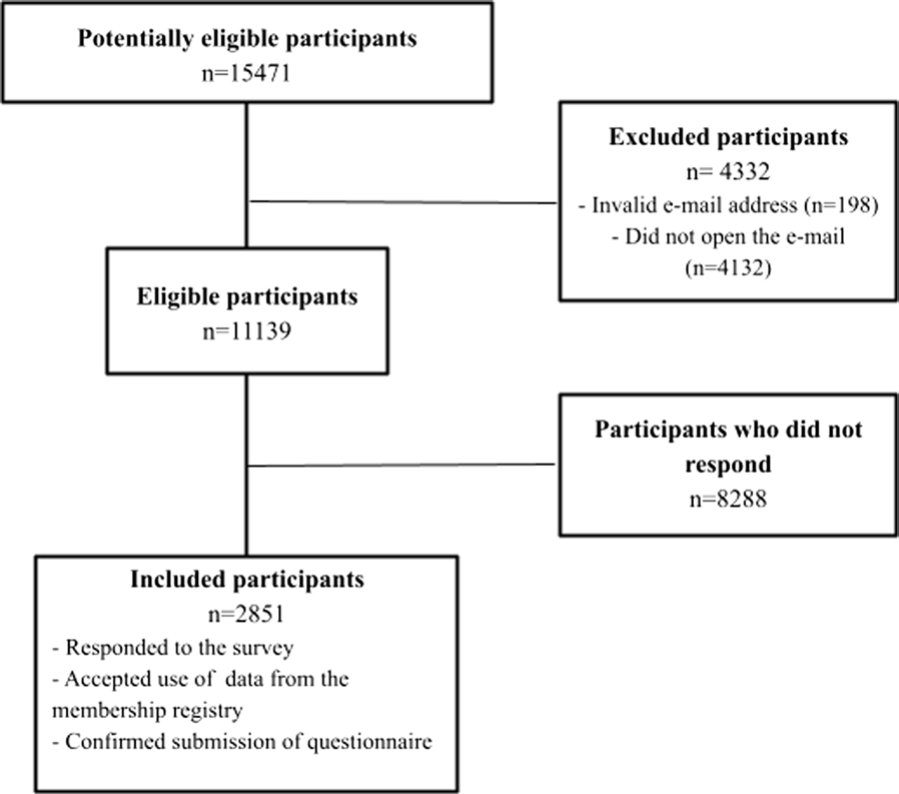
Flow chart of participants

Of the 11,139 eligible members, 2851 (26%) opened the email, accepted the invitation, completed the survey, consented to the linkage to the membership registry, and were available for statistical analyses (Fig. 1). Descriptive statistics of the study population appear in Table 1.

**Table 1:** Characteristics of the 2851 long-term members in the study population at the time of survey participation in June 2018

	Proportion of participants according to number of days with visits				
Variable	0 (n = 70)	1–57 (n = 924)	58–117 (n = 923)	118–543 (n = 934)	Total (n = 2851)
Sex					
Female	60	65	63	59	62
Male	40	35	37	41	38
Age					
< 20 years	0	0	0	1	1
20–29 years	1	7	9	13	10
30–39 years	16	25	17	19	20
40–49 years	31	29	24	24	26
50–59 years	27	22	26	24	24
60–69 years	20	12	14	11	13
≥ 70 years	4	5	10	8	8
Education^a^					
Compulsory	0	1	2	1	1
Middle	21	18	19	25	21
Higher	79	81	79	73	78
Employment					
Full time work	81	79	71	70	74
Part time work	4	7	7	8	7
Student	1	2	3	4	3
Not in work ^b^	13	11	19	17	15
Other	0	1	1	1	1

^a^Education: Compulsory (primary school and middle school graduation or lower), middle (high school graduation (duration 1–2 years), high school graduation (duration three years), certificate of apprenticeship (duration four years)), and higher (college/university graduation)

^b^Not in work: occupational rehabilitation, unemployed or laid off, disability benefits or retired

The number of days with visits to the fitness center during the 18 months before answering the survey ranged from 0 to 543 (mean of 97 (SD 73), with a median of 82 days (interquartile range 43 to 135)). Ranging from 0 to 100, goal achievement had a mean of 68 (*SD* = 20) and a median of 71 (interquartile range = 57–80).

Overall, less use of the fitness center was associated with poorer goal achievement (Table 2). Participants in the lowest third category of number of days with visits to a center had a geometric mean of 36 on the inverted goal achievement scale (i.e., higher values correspond to lower goal achievement), whereas those in the highest third (reference group) had a geometric mean of 19 (ratio of geometric means = 1.9; 95% CI 1.8–2.1). Similarly, the ratio of geometric means comparing members with less than four 3-month periods of visits (geometric mean of 38) to the reference group with visits in all six 3-month periods (geometric mean of 24) was 1.6 (95% CI 1.5–1.8). The number of group activity bookings showed similar associations, with lower goal achievement in those with fewer bookings. There was no association between the number of bookings with a fitness trainer and mean goal achievement score.

**Table 2:** Fitness center use during 18 months associated with subsequent mean goal achievement score among 2851 long-term fitness members

Fitness center useduring 18 months	No. of persons (%)	Mean goal achievement score^a^	Crude ratio^b^	Adjusted^c^ ratio^b^	95% CI^d^
Number of days with visits (thirds)					
None	70 (3)	30	1.6	1.6	1.3–1.9
Low (1–57)	923 (32)	36	1.9	1.9	1.8–2.1
Medium (58–117)	924 (32)	25	1.3	1.3	1.2–1.4
High (118–543)	934 (33)	19	1.0	1.0	Ref
Number of three-month periods with visits					
< 4	268 (9)	38	1.6	1.6	1.5–1.8
4–5	441 (16)	31	1.3	1.3	1.2–1.4
6	2142 (75)	24	1.0	1.0	Ref
Number of group activity bookings (thirds)					
None	638 (22)	29	1.5	1.5	1.4–1.7
Low (1–26)	744 (26)	31	1.7	1.7	1.5–1.8
Medium (27–80)	729 (26)	27	1.5	1.5	1.3–1.6
High (81–550)	740 (26)	19	1.0	1.0	Ref
Number of fitness trainer bookings					
None	2391 (84)	26	1.0	1.0	Ref
1	273 (10)	25	0.9	0.9	0.8–1.0
≥ 2	187 (7)	24	0.9	0.9	0.8–1.0

CI = confidence interval

^a^Geometric mean from an inverted VAS (0–100). Higher score indicates lower self-reported goal achievement

^b^Ratio between geometric means

^c^Adjusted for age < 30, 30–39, 40–49, 50–59, 60–69 and ≥ 70 years), higher education (yes, no), and sex (man, woman)

^d^95% CI for adjusted ratio

Correspondingly, the use of fitness centers was inversely associated with the proportion who reported low goal achievement on the binary outcome variable (Table 3). Participants in the lowest category of days with visits (OR = 8.5; 95% CI 6.3–11.4) and visits in less than four 3-month periods (OR = 4.7; 95% CI 3.6–6.2) were more likely to report low goal achievement than those in the categories with the highest number of days with visits and visits during all 3-month periods (reference groups), respectively. Members in the lowest third of group activity bookings were more likely to report low goal achievement than those in the upper third (OR = 5.1; 95% CI 3.7–7.1). There was some evidence that participants who reported two or more bookings with a fitness trainer were less likely to report low goal achievement than those without such bookings (OR = 0.6; 95% CI 0.4–1.0).

**Table 3:** Fitness center use during 18 months associated with subsequent goal achievement among 2851 long-term fitness members. Crude and adjusted odds ratio for reporting low goal achievement^a^

Fitness center use during 18 months	No. of persons	No. of cases	Crude odds ratio	Adjusted odds ratio^b^	95% CI^c^
Number of days with visits (thirds)					
None	70	22	6.7	6.9	3.9–12.2
Low (1–57)	923	339	8.4	8.5	6.3–11.4
Medium (58–117)	924	121	2.2	2.2	1.6–3.1
High (118–543)	934	60	1.0	1.0	Ref
Number of three-month periods with visits					
< 4	268	117	4.7	4.7	3.6–6.2
4–5	441	124	2.4	2.4	1.9–3.0
6	2142	301	1.0	1.0	Ref
Number of group activity bookings (thirds)					
None	638	165	4.5	5.5	3.9–7.8
Low (1–26)	744	199	4.7	5.1	3.7–7.1
Medium (27–80)	729	125	2.7	2.8	2.0–3.9
High (81–550)	740	53	1.0	1.0	Ref
Number of fitness trainer bookings					
None	2391	464	1.0	1.0	Ref
1	273	54	1.0	1.0	0.8–1.5
≥ 2	187	24	0.6	0.6	0.4–1.0

CI: Confidence interval

^a^Low goal achievement was defines as scores between 0 and 50 on the VAS-scale

^b^Adjusted for age (< 30, 30–39, 40–49, 50–59, 60–69 and ≥ 70 years), higher education (yes, no), and sex (man, woman)

^c^95% CI for adjusted ratio

## Discussion

In this registry- and survey-based longitudinal study among long-term members of fitness centers, a higher number of days with fitness center visits and regular use of fitness centers during and 18 month period were associated with higher subsequent self-reported achievement of exercise goals. Regarding the use of services within the fitness centers, the number of group activity bookings had associations similar to those observed for number of visits, whereas number of bookings with a fitness trainer was not consistently associated with goal achievement.

This study provides new knowledge on the association between the use of fitness centers and goal achievement. Goal achievement has typically been measured on whether concrete goals, such as number of daily steps [16, 17], minutes of physical activity per week, and changes in body weight [18], are met. However, such concrete measures omits the overall experiences of own goal achievement. Thus, the use of a single question to measure overall goal achievement could be used in other studies either alone or in addition to measures of concrete goals.

Visiting a fitness center at least once every third month was associated with higher goal achievement. Research has shown that few members use fitness centers regularly without periods of relapse [6]. Thus, measuring the regularity of visits, as done in this study, adds important knowledge about the maintenance of physical activity over time, which also is relevant for public health [19]. We have not found any other studies measuring regularity in this manner. Other studies tend to be cross sectional without clearly distinguishing frequency from regularity [20, 21].

It was also found that frequent fitness center visits were associated with higher goal achievement. A Danish report using cross-sectional questionnaire data asked about "regular exercise several times a week" and found that a higher proportion of members who answered yes to this also reported to achieve their exercise goals than members reporting irregular exercise (18% vs. 5%) [20]. In a published cross-sectional survey from Portugal measuring the association between self-reported weekly frequency of fitness center use and satisfaction, found no association [21].

Given the longitudinal design of our study, it seems fair to state that current available evidence points to frequent and regular fitness center visits is associated with higher goal achievement among long-term members. However, the bi-directional nature of goal achievement and the use of fitness centers needs to be kept in mind. Since we do not know the initial goal achievement status, the members` perceived goal achievement level at the study`s start might have caused the members` fitness center use, instead of the other way around.

Thus, future longitudinal studies should assess goal achievement both before and after measuring the use of fitness centers.

In addition to visits, we also measured the use of fitness center services, group activity and fitness trainer bookings to further understand which aspects of the use of fitness centers were associated with goal achievement. Among our results, a higher number of group activity bookings was associated with higher self-reported goal achievement.

In a qualitative study among long-term members, joining group activities was told to demand less self-motivation, help to commit more to exercise, and induce more vigorous exercise than self-training [13]. Other studies have found that participating in group activities can improve health-related quality of life [22], enhance aerobic capacity [23], and affect physical activity behavior [24], which might help to explain why members using that specific service reported higher goal achievement.

Previous research has revealed that one-to-one support can contribute to regular fitness center use [25]. In addition, help and instructions given by fitness trainers have been described positively by long-term members in a qualitative study and said to increase their understanding of using the fitness center as a means for physical activity [13]. However, in the current study fitness trainer bookings were not consistently associated with members` subsequent goal achievement. One reason may be that very few members used that specific service and that most had only one booking during the 18-month period.

### Strengths and limitations

The longitudinal data and large number of participants are considerable strengths of the current study. In contrast to self-reported activity, registry data provides objectively measured activities and avoids measurement errors [26]. However, this only provided data on number of activities and not the length of or type of activity. Furthermore, the study only focused on activities related to the fitness center, and did not measure the influence of daily life, other physical activities, diet or other external factors.

The reliability or validity of the dependent variable, goal achievement, was not assessed in advance of the study [27]. Some members with no visits during the 18-month study period reported high goal achievement (i.e., ≥ 50 on the 0–100 VAS scale). A possible explanation is that members who scored high on goal achievement without any visits were satisfied with their total activity level and did not differentiate activity inside and outside the fitness center. It is also possible that members were satisfied with the fitness center as an arena for physical activity but without using it during the last 18 months and thus reported high goal achievement. However, the dependent variable was measured with a single question, and the findings were in the expected direction, which indicates that the variable measured what was intended.

Another limitation was that only 26% of eligible members who opened the invitation email chose to participate. Low response rates can increase the risk of selection bias and reduce external validity. Furthermore, the participants were all members at one fitness center chain. Caution should therefore be used when generalizing the findings to other fitness center members.

## Conclusion

In this longitudinal study on long-term fitness center members, more days with fitness center visits and regular use of the fitness centers during an 18-month period were associated with higher subsequent self-reported exercise goal achievement.

## Data Availability

The anonymized data files are available from the corresponding author on reasonable request.

## Abbreviations

VAS: Visual analogue scale
OR: Odds ratio
CI: Confidence interval
SD: Standard deviation
